# Resection and reconstruction of pancreatic artery aneurysms caused by the compression of the celiac trunk by the median arcuate ligament: a report of two cases

**DOI:** 10.1186/s40792-021-01247-y

**Published:** 2021-07-16

**Authors:** Hideaki Uchiyama, Sosei Kuma, Mayumi Ishida, Eiji Tsujita, Yoshinari Nobuto, Yuta Kasagi, Keita Natsugoe, Takehiko Aoyagi, Tomohiro Iguchi, Hiroyuki Itoh

**Affiliations:** 1 Department of Surgery, National Hospital Organization Fukuokahigashi Medical Center, Koga, 811-3195 Japan; 2 Department of Surgery, Saiseikai Fukuoka General Hospital, Fukuoka, 810-0001 Japan

**Keywords:** Median arcuate ligament, Pancreatic artery aneurysm, Rupture, Resection and reconstruction

## Abstract

**Background:**

Some patients with the compression of the celiac trunk by the median arcuate ligament (MAL) suffer pancreatic artery aneurysms (PAAs) due to excessive blood flow from the superior mesenteric artery. These aneurysms are in peril because they are prone to rupture irrespective of size. Here, we present two cases of resection and reconstruction of PAAs caused by the compression of the celiac trunk by the MAL.

**Case presentation:**

Patient 1 was a 44-year-old man who was first diagnosed to have a visceral artery aneurysm with a diameter of 4 cm accidentally found by ultrasound examination at a regular medical check-up. Contrast-enhanced CT revealed the compression of the celiac trunk by the MAL and a PAA originating from the first jejunal artery. First, laparoscopic excision of the MAL followed by a stent placement into the celiac trunk was performed. Although the stent was patent, the PAA still grew. The patient underwent resection and reconstruction of the PAA. Reconstruction of the pancreatic arterial arcade was needed because clamping of the inferior pancreaticoduodenal artery (IPDA) resulted in disappearance of the hepatic arterial blood flow. The follow-up CT 2 years and 9 months after the operation revealed no recurrence of aneurysms and the patent anastomosis. Patient 2 was a 68-year-old man who presented with an epigastric pain. Contrast-enhanced CT revealed the compression of the celiac trunk by the MAL and a PAA approximately 6 cm in diameter originating from the IPDA. The PAA was surrounded by a relatively low-intensity area, suggesting impending rupture of the PAA. The patient underwent resection and reconstruction of the PAA under an emergency situation. Reconstruction of the pancreatic arterial arcade was needed because clamping of the inflow IPDA resulted in disappearance of the hepatic blood flow. The follow-up CT 1 year and 8 months after the operation revealed no recurrence of aneurysms and the patent anastomosis.

**Conclusions:**

Although long-term follow-up is needed, resection and reconstruction is one of the therapeutic choices for PAAs caused by the compression of the celiac trunk by the MAL in order to prevent catastrophic aneurysm rupture.

## Background

In patients with the compression of the celiac trunk by the median arcuate ligament (MAL), excessive blood flow occurs in pancreatic arteries such as the inferior pancreaticoduodenal artery (IPDA) or the gastroduodenal artery (GDA) in order to maintain arterial blood from the superior mesenteric artery (SMA) to celiac trunk branches. Sometimes, excessive blood flow causes aneurysm formation in pancreatic arteries [1]. These aneurysms are in peril because they are prone to rupture irrespective of size [2]. Here, we present two patients with a pancreatic artery aneurysm (PAA) which was treated by resection and reconstruction.

## Case presentation

### Patient 1

**Fig. 1:**
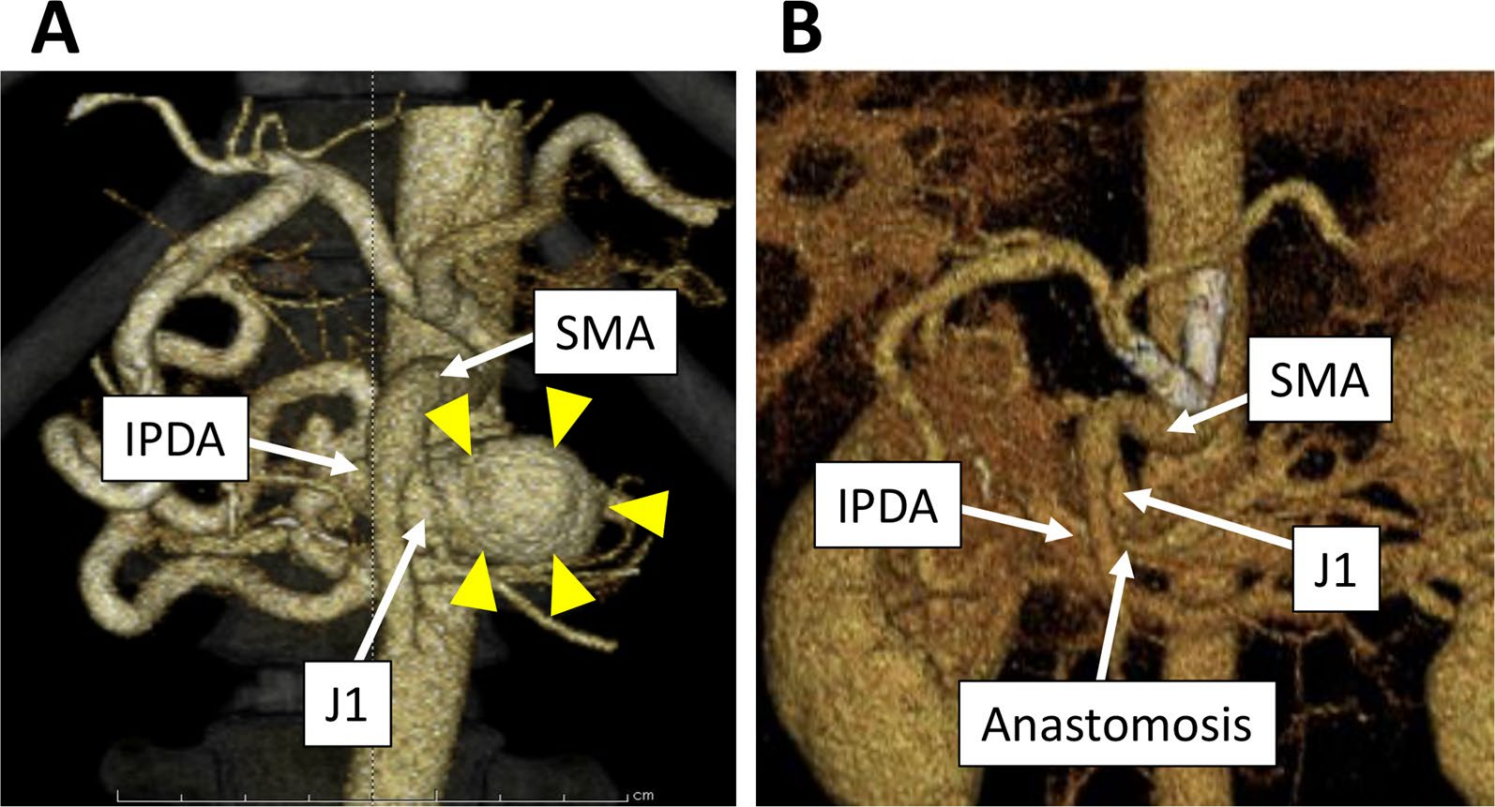
3D-CT finding of the pancreatic arterial arcade in Patient 1**.** **A** Preoperative 3D-CT. The arrowheads indicate the pancreatic artery aneurysm. **B** 3D-CT 2 years and 9 months after the operation. *IPDA* inferior pancreaticoduodenal artery, *J1* first jejunal artery, *SMA* superior mesenteric artery

Patient 1 was a 44-year-old man who was first diagnosed to have a visceral artery aneurysm with a diameter of 43 mm accidentally found by ultrasound examination at a regular medical check-up. He was on anti-hypertensives and lipid-lowering drugs. There was no other remarkable past medical history. Contrast-enhanced CT revealed the compression of the celiac trunk by the MAL and a PAA originating from the first jejunal artery (J1) (Fig. 1A).

First, the patient underwent laparoscopic excision of the MAL in order to release the compression of the celiac trunk. The follow-up angiography revealed that the blood flow of the GDA was retrograde and that the arterial blood to the liver, the stomach, and the spleen were completely supplied by the SMA via the GDA. A stent was placed at the celiac trunk in an attempt to increase the blood flow of the celiac trunk. A coil embolization of the aneurysm was abandoned because of anatomical difficulties. The follow-up CT revealed the gradually growing PAA. The patient was finally willing to undergo resection and reconstruction of the aneurysm which seemed to rupture sooner or later.

**Fig. 2:**
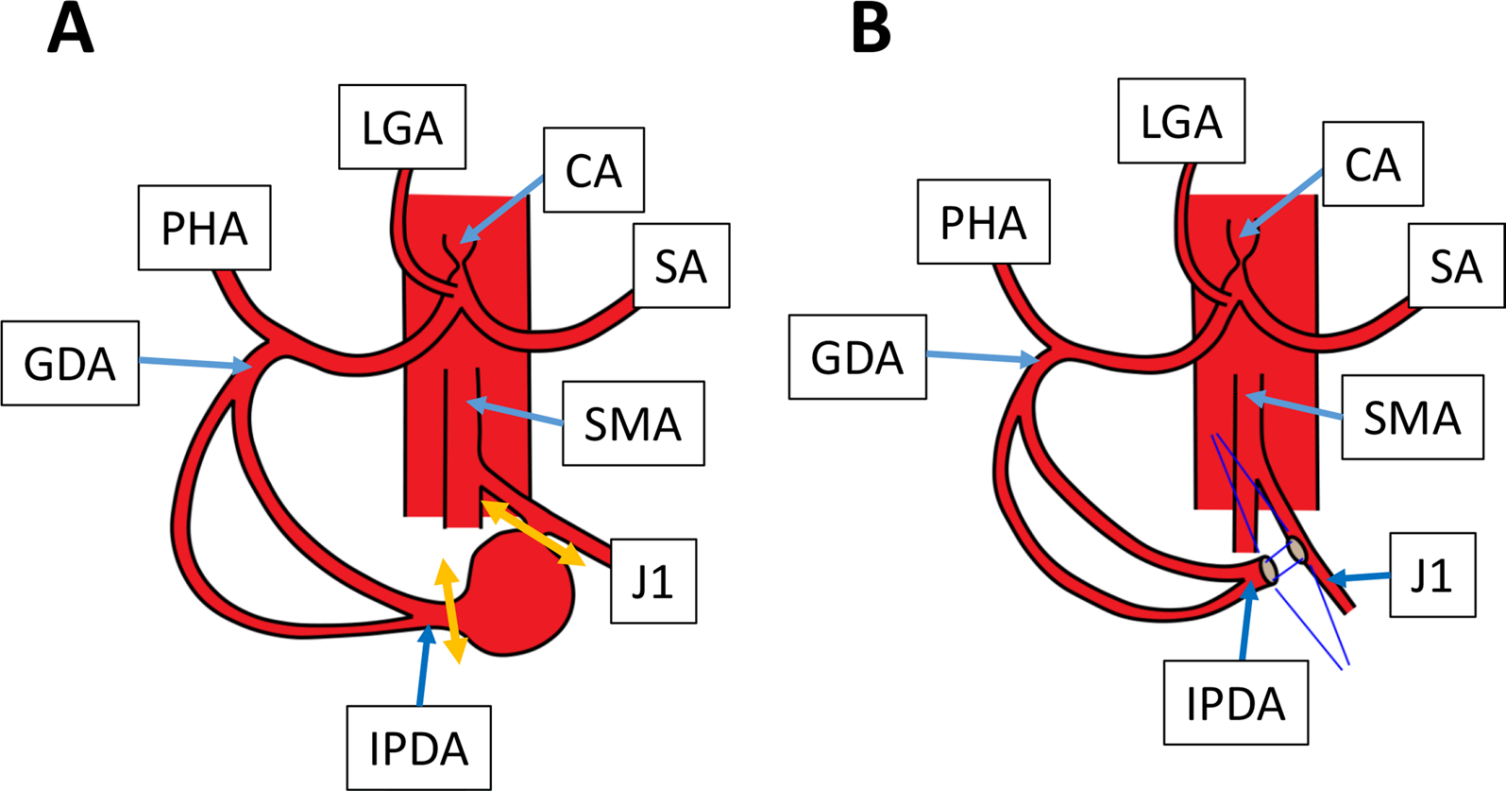
Schematic diagrams of resection and reconstruction of the pancreatic artery aneurysm in Patient 1. **A** Resection of the aneurysm. The double arrows indicate the resecting points. **B** Reconstruction of the pancreatic arterial arcade. *CA* celiac artery, *GDA* gastroduodenal artery, *IPDA* inferior pancreaticoduodenal artery, *J1* first jejunal artery, *LGA* left gastric artery, *PHA* proper hepatic artery, *SA* splenic artery, *SMA* superior mesenteric artery

The abdomen was entered by an upper midline incision. The aneurysm was located near the ligament of Treitz (Fig. 2). The SMA, the IPDA, the J1, the proper hepatic artery (PHA), and the GDA were controlled with tapes. First, the GDA was clamped in order to test whether the hepatofugal flow from the celiac trunk was restored. Although the stent placed in the celiac trunk proved to be patent by the preoperative CT, the attempt was not successful. The retrograde blood flow from the SMA via the GDA proved to be vital to the liver, the stomach, and the spleen. Next, the J1 was clamped in order to test whether this artery could be sacrificed in an attempt to simply reconstruct the arteries by an end-to-end anastomosis. Because the color of the proximal jejunum was darkened after this artery was clamped, this artery could not be sacrificed. The aneurysm was resected just below the IPDA branch of the J1 (Fig. 2A). The flow of the IPDA was restored by an end-to-side anastomosis between the IPDA and the J1 (Fig. 2B), as previously reported [3]. The anastomotic procedure was completely done on the left side of the SMA. The posterior walls were intraluminally sutured by a continuous 7-0 non-absorbable suture and the anterior walls were sutured by an over-and-over continuous 7-0 non-absorbable suture.

The operation time was 308 min. and the intraoperative blood loss was 220 ml. The postoperative course was uneventful and he was discharged from the hospital on the postoperative day 7. His blood pressure was strictly controlled by anti-hypertensives. The follow-up CT 2 years and 9 months after the operation revealed no recurrence of aneurysms and the patent anastomosis (Fig. 2B).

### Patient 2

**Fig. 3:**
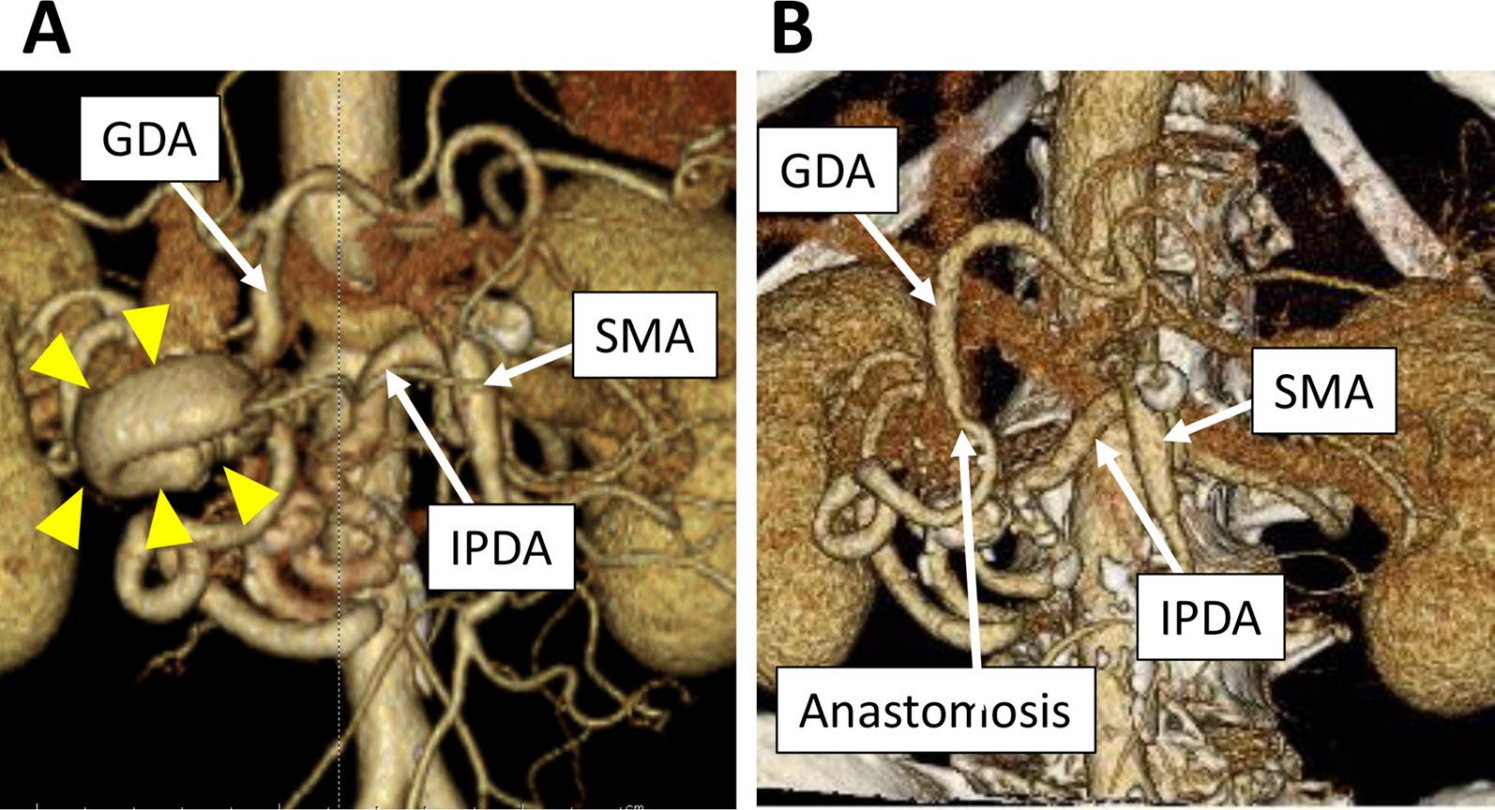
CT finding of the pancreatic arterial arcade in Patient 2. **A** Preoperative 3D-CT. The arrowheads indicate the pancreatic artery aneurysm. **B** 3D-CT 1 year and 8 months after the operation. *GDA* gastroduodenal artery, *IPDA* inferior pancreaticoduodenal artery, *SMA* superior mesenteric artery

Patient 2 was a 68-year-old man who presented with an epigastric pain. He was on anti-hypertensives. There was no other remarkable past medical history. Contrast-enhanced CT revealed that the celiac trunk was compressed by the MAL and a PAA approximately 6 cm in diameter. The PAA originated from the anterior IPDA and was surrounded by a relatively low-intensity area suggesting impending rupture of the PAA (Fig. 3A). An emergency operation was conducted.

**Fig. 4:**
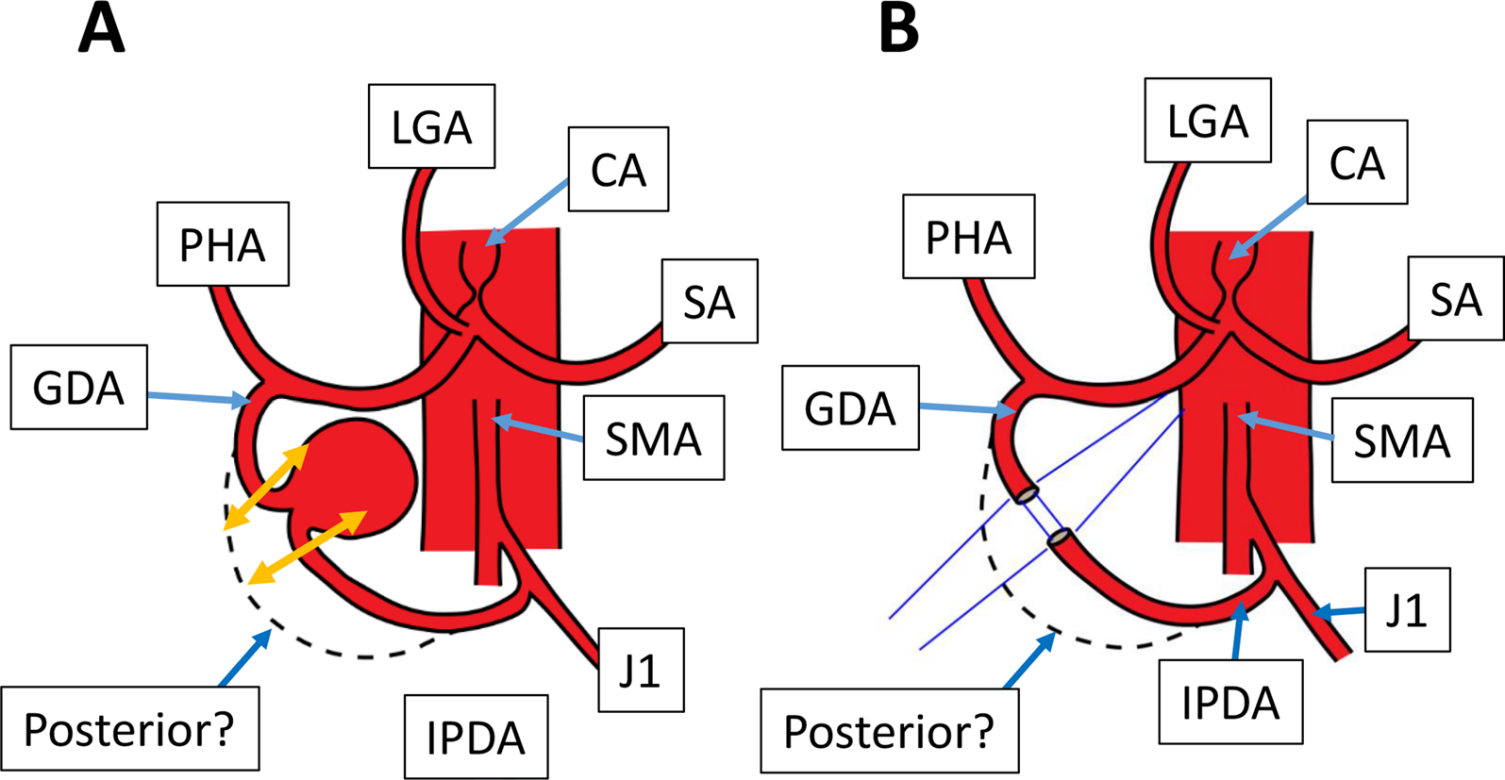
Schematic diagrams of resection and reconstruction of the pancreatic artery aneurysm in Patient 2. **A** Resection of the aneurysm. The double arrows indicate the resecting points. **B** Reconstruction of the pancreatic arcade. *CA* celiac artery, *GDA* gastroduodenal artery, *IPDA* inferior pancreaticoduodenal artery, *J1* first jejunal artery, *LGA* left gastric artery, *PHA* proper hepatic artery, *SA* splenic artery, *SMA* superior mesenteric artery

The abdomen was entered by an upper midline incision. The PAA was located in front of the pancreas head and surrounded by the dark-colored omentum. By carefully dissecting the omentum, the PAA was exposed. The PAA’s inflow (the anterior IPDA) and outflow (the GDA) were controlled with tapes (Fig. 4). When the PAA’s inflow was clamped, the pulses of the PHA disappeared, suggesting that the hepatic blood flow was completely dependent on this clamped inflow and that the reconstruction of the pancreatic arterial arcade was mandatory although we did not confirm the presence of the posterior branch of the IPDA. The aneurysm was resected by cutting the anterior IPDA and the GDA near the aneurysm (Fig. 4A). The pancreatic arterial arcade was reconstructed by an end-to-end anastomosis between the anterior IPDA and the GDA (Fig. 4B). The MAL was left untouched because of the emergency situation.

The operation time was 260 min. and the intraoperative blood loss was 184 ml. The postoperative course was uneventful and he was discharged from the hospital on the postoperative day 16. His blood pressure was strictly controlled by anti-hypertensives. The follow-up CT 1 year and 8 months after the operation revealed no recurrence of aneurysms and the patent anastomosis (Fig. 3B).

## Discussion

The probability of rupture is said to be higher for PAAs than for other visceral artery aneurysms [4]. Therefore, simple follow-up would be dangerous just because of its small size. So far, there has been no therapeutic guideline or strategy for PAAs caused by MAL compression to the celiac trunk. Four main therapeutic options have been reported, namely MAL division [1, 5], stenting into the celiac trunk [6, 7], coil embolization into the aneurysm [2, 8–11], and resection and reconstruction of aneurysms [12, 13].

First, MAL division has been reported to be effective in some cases [1, 5]. MAL division can be carried out not only by an open surgery [1] but also by a laparoscopic surgery [5]. By dividing the MAL, the compression to the celiac trunk could be released, then the antegrade flow in the celiac trunk could be restored. As a result, the excessive blood flow in the pancreatic arcade is expected to be normalized, which would result in PAA shrinking. In Patient 1, this attempt failed. We presume that it is difficult to reverse retrograde strong blood flow that has been flowing in such a way for a long time. In Patient 2, this attempt was hard to be conducted because of its emergency situation—impending rupture of the PAA. We will continue follow-ups of Patient 2. When any new aneurysms appear, we will try to resect the MAL.

Second, some authors reported the efficacy of stenting into the celiac trunk [6, 7]. The aim of stenting into the celiac trunk is also to restore the antegrade flow in the celiac trunk, which would result in normalization of the blood flow in the pancreatic arcade and then PAA shrinking. Again, this attempt failed in Patient 1, although the stent was proved to be patent. As above-mentioned, it is considered difficult to reverse retrograde strong blood flow that has been flowing in such a way for a long time. This attempt was hard to be conducted in Patient 2 because of the emergency situation.

Third, there has been several reports demonstrating the effectiveness of coil embolization [2, 8]. Coil embolization may have a risk of shutting down the vital arterial blood flow to the liver, the pancreas, and the stomach. Some authors reported that coil embolization could be safely performed without celiac revascularization by MAL release [9, 10]. Others stated that patients who had at least one visible collateral pathway between the celiac branches and the SMA branches which was free from aneurysms were treated with coil embolization [11]. As shown in Fig. 2, 4, coil embolization in our cases must have resulted in complete shutdown of the pancreatic arterial arcade which is vital to the liver, etc., because intraoperative clamping the IPDA resulted in the disappearance of PHA flow. If there are any other collateral flows from the SMA to branches of the celiac trunk, coil embolization could be performed safely. Otherwise, this attempt would be dangerous—serious arterial ischemia to the liver.

Fourth, resections and reconstructions of PAAs are considered effective in some cases [12, 13]. Ritter [12] and Nishiyama [13] reported arterial flow reconstruction of the celiac trunk branches by bypass creation between the aorta/the iliac artery and the GDA. We performed simple resection and reconstruction of PAAs caused by the MAL compression to the celiac trunk. The operative outcomes were well and there has been no recurrence of aneurysms so far. This procedure should be a choice for such PAAs if a surgeon has a technique of reconstructing visceral arteries. In our two cases, clamping the IPDA resulted in disappearance of PHA’s pulsation. After the reconstruction, the pulsation revived. Only a surgical intervention can make a surgeon confident that there is sufficient arterial flow to the liver by directly palpating the arteries. Intervention radiology or image analyses cannot do so. However, there exists PAAs that cannot be resected and reconstructed. When a PAA is located deep in the pancreas parenchyma, simple resection and reconstruction might be impossible. If there are multiple PAAs, reconstruction might also be impossible because of anatomical restrictions. Meticulous preoperative evaluation and planning are mandatory when considering such operations.

## Conclusion

Although long-term follow-up is needed, resection and reconstruction is one of the therapeutic choices for PAAs caused by MAL compression to the celiac trunk in order to prevent catastrophic aneurysm rupture. Precise preoperative planning is needed because there is a possibility of demanding a reconstruction of the pancreatic arterial arcade.

## Data Availability

Not applicable.

## Abbreviations

CA: Celiac artery
GDA: Gastroduodenal artery
IPDA: Inferior pancreaticoduodenal artery
J1: First jejunal artery
LGA: Left gastric artery
MAL: Median arcuate ligament
PAA: Pancreatic artery aneurysm
PHA: Proper hepatic artery
SA: Splenic artery
SMA: Superior mesenteric artery
SMV: Superior mesenteric vein
